# Supplementary material for: DIS3 mutations enhance AID-driven translocations during B-cell activation, promoting transformation to multiple myeloma
Source: Nat Commun. 2026 Mar 14;17:3976. doi: 10.1038/s41467-026-70386-3 (PMC13133341; doi:10.1038/s41467-026-70386-3)
Supplement: Supplementary file 1 — Supplementary Information [file 41467_2026_70386_MOESM1_ESM.pdf]

Supplementary information:

DIS3 Mutations Enhance AID-Driven Translocations During B-Cell Activation, Promoting Transformation to Multiple Myeloma

Tomasz M. Kuliński[1, 2,*], Olga Gewartowska[3, 4], Mélanie Mahé[5,6], Karolina Kasztelan[1,2], Nina Durys[2], Anna Stroynowska-Czerwińska[7], Marta Jedynak-Slyvka[8,9,†], Ewelina P. Owczarek[1], Debadeep Chaudhury[7], Marcin Nowotny[8], Aleksandra Pękowska[7], Bertrand Séraphin[5,6], and Andrzej Dziembowski[1,2, 3,*]

Affiliations:

[1] Laboratory of RNA Biology, International Institute of Molecular and Cell Biology, 4 Trojdena Street, Warsaw, 02-109, Poland. [2] Institute of Biochemistry and Biophysics, Polish Academy of Sciences, Pawinskiego 5a, Warsaw, 02-106, Poland. [3] Faculty of Biology, University of Warsaw, Pawinskiego 5a, Warsaw, 02-106, Poland. [4] Genome Engineering Facility, International Institute of Molecular and Cell Biology, 4 Trojdena Street, Warsaw, 02-109, Poland. [5] Institut de Génétique et de Biologie Moléculaire et Cellulaire, Illkirch, France; Centre National de Recherche Scientifique, UMR 7104, Illkirch, France; [6] Institut National de Santé et de Recherche Médicale, U964, Illkirch, France; Université de Strasbourg, Illkirch, France. [7] Dioscuri Center for Chromatin Biology and Epigenomics, Nencki Institute of Experimental Biology, Polish Academy of Sciences, 3 Pasteur Street, Warsaw, 02-093, Poland. [8] Laboratory of Protein Structure, International Institute of Molecular and Cell Biology, 4 Trojdena Street, Warsaw, 02-109, Poland. [9] International Institute of Molecular Mechanisms and Machines, Polish Academy of Sciences, Flisa 6, 02-247 Warsaw, Poland † Current adress: Technical University of Denmark, Centre for Diagnostics, Department of Health Technology, Henrik Dams Allé, 2800 Kgs. Lyngby, Denmark

*Correspondence to: adziembowski@iimcb.gov.pl, tkulinski@iimcb.gov.pl

SUPPLEMENTAL DATA

- **Supplementary Figure S1**
- **Supplementary Figure S2**
- **Supplementary Figure S3**
- **Supplementary Figure S4**
- **Supplementary Figure S5**
- **Supplementary Figure S6**
- **Supplementary Figure S7**
- **Supplementary Figure S8**
- **Supplementary Figure S9**
- **Supplementary Figure S10**
- **Supplementary Tables**
- **Description of Supplementary Data**

SUPPLEMENTARY FIGURES

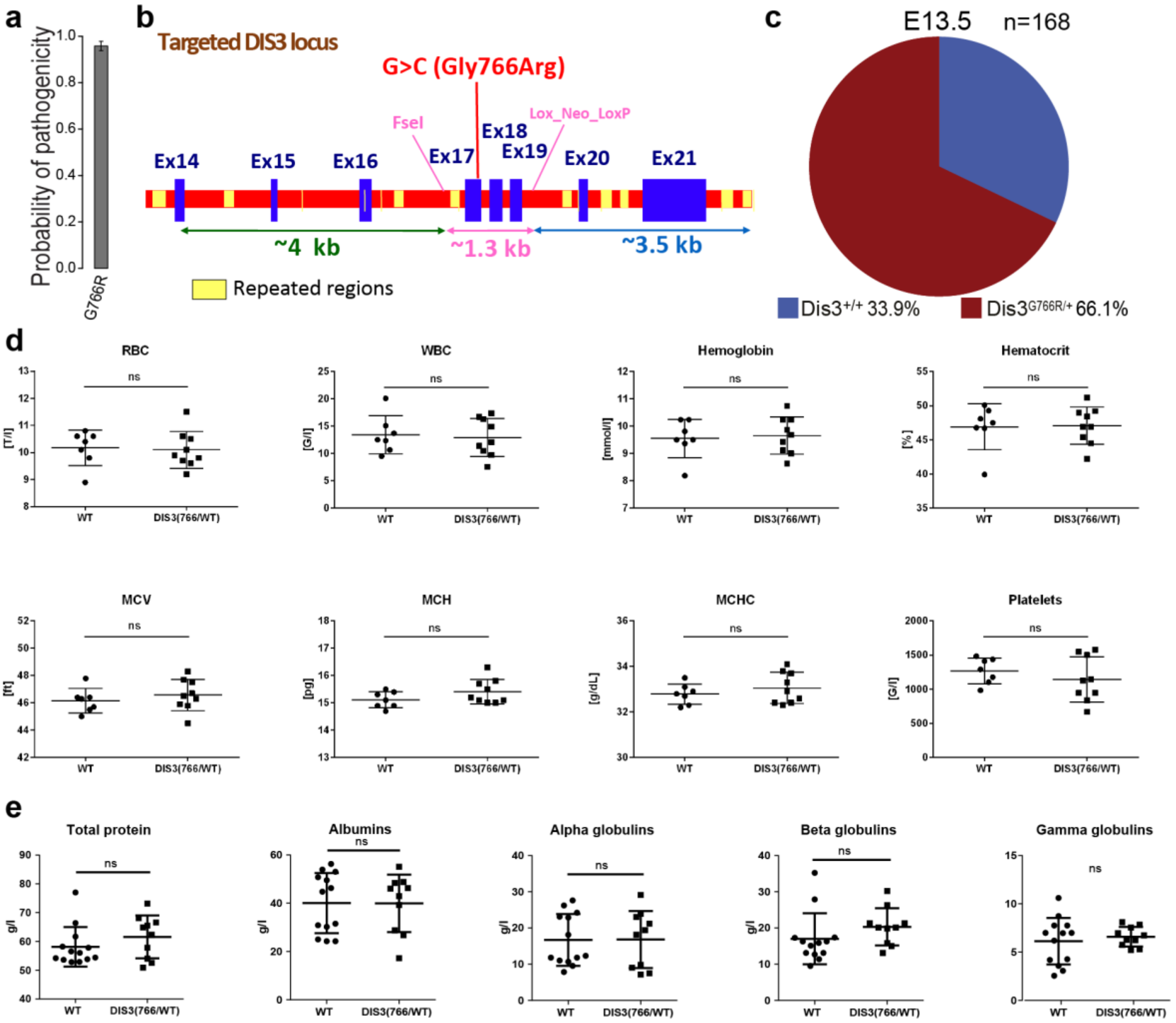

Supplementary Figure S1. DIS3^{G766R} allele leads to early embryonic lethality in homozygous mice, but heterozygous mice do not display any obvious abnormalities. a Prediction of G766R Dis3 variant pathogenicity by PON-P2. Error bars represent standard deviation. **b** Strategy for the generation of Dis3^{G766R} knock-in mice. **c** Frequencies of offspring of Dis3$^{G766R/+}$ × Dis3$^{G766R/+}$ mattings on mouse embryonic day 13.5 (E13.5) of development with indicated genotypes. Dis3$^{G766R/+}$ mice do not show any abnormalities in **d** blood morphology or **e** disruptions of protein fractions in serum protein electrophoresis.

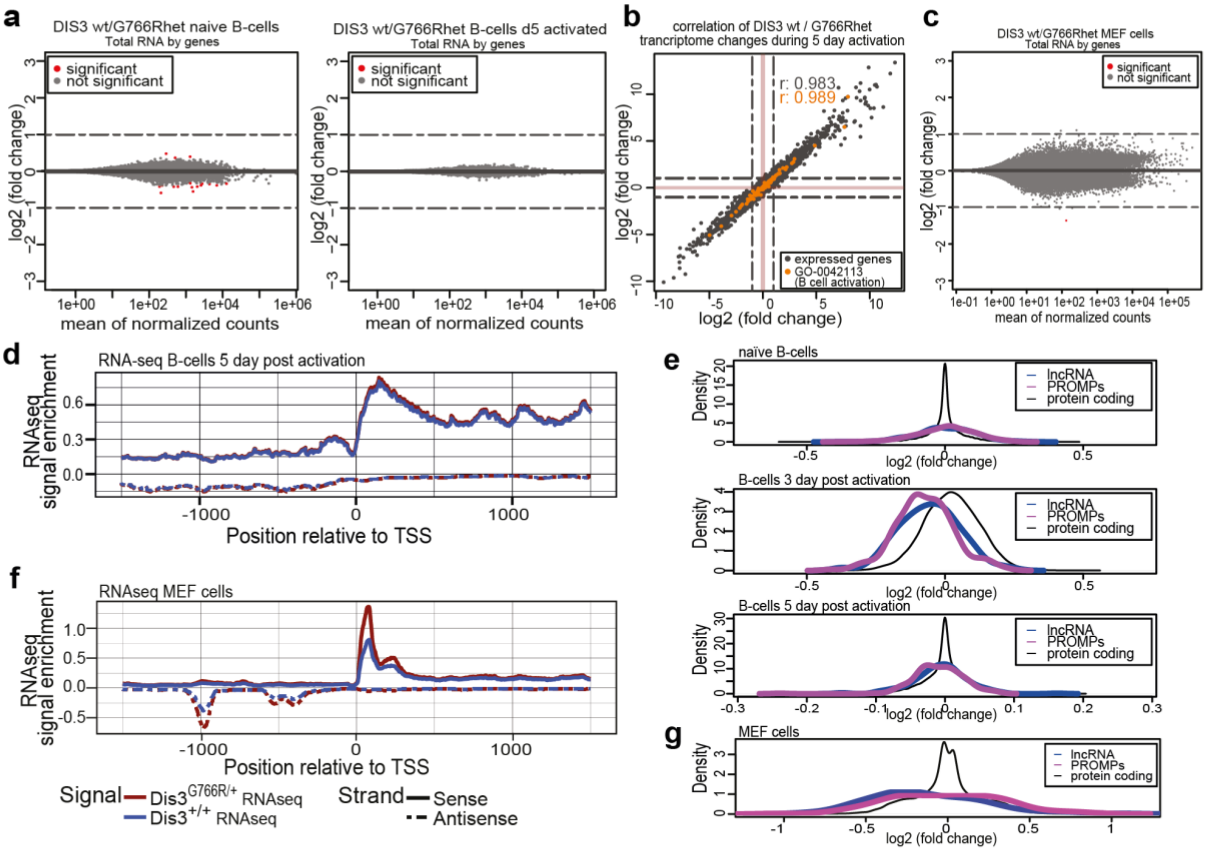

Supplementary Figure S2. In vitro activated Dis3$^{G766R/+}$ B-cells present a molecular phenotype compared to WT B-cells. **a** MA plot comparing the transcriptome of primary Dis3$^{G766R/+}$ and Dis3$^{+/+}$ B cells naïve and *in vitro* activated for 5 days (n=3 for each genotype). **b** Correlation of transcriptome changes in the DIS3$^{G766R/+}$ and DIS3$^{+/+}$ during *in vitro* activation for 5 days. **c** MA plot showing comparisons of the transcriptome of WT MEFs with Dis3G766R/+ MEFs. The dashed lines on the plot represent a threshold of 2-fold change. **d** Meta-analyses of RNA seq signal over TSS of expressed genes day 5 activated B cells **e** The accumulation of PROMPT and long non-coding transcripts (lncRNA) in naïve, day 3, and day 5 in vitro activated B-cells. **f** Meta-analyses of RNA seq signal over TSS of expressed genes in MEF cells. **g** The distribution of log2 of fold change values shows a moderate accumulation of long non-coding transcripts (lncRNA) in DIS3$^{G766R/+}$ MEF cells, peaking at around 25% (~1.25 fold change). (Statistics: two-sided Wald test with Benjamini–Hochberg multiple-testing correction.)

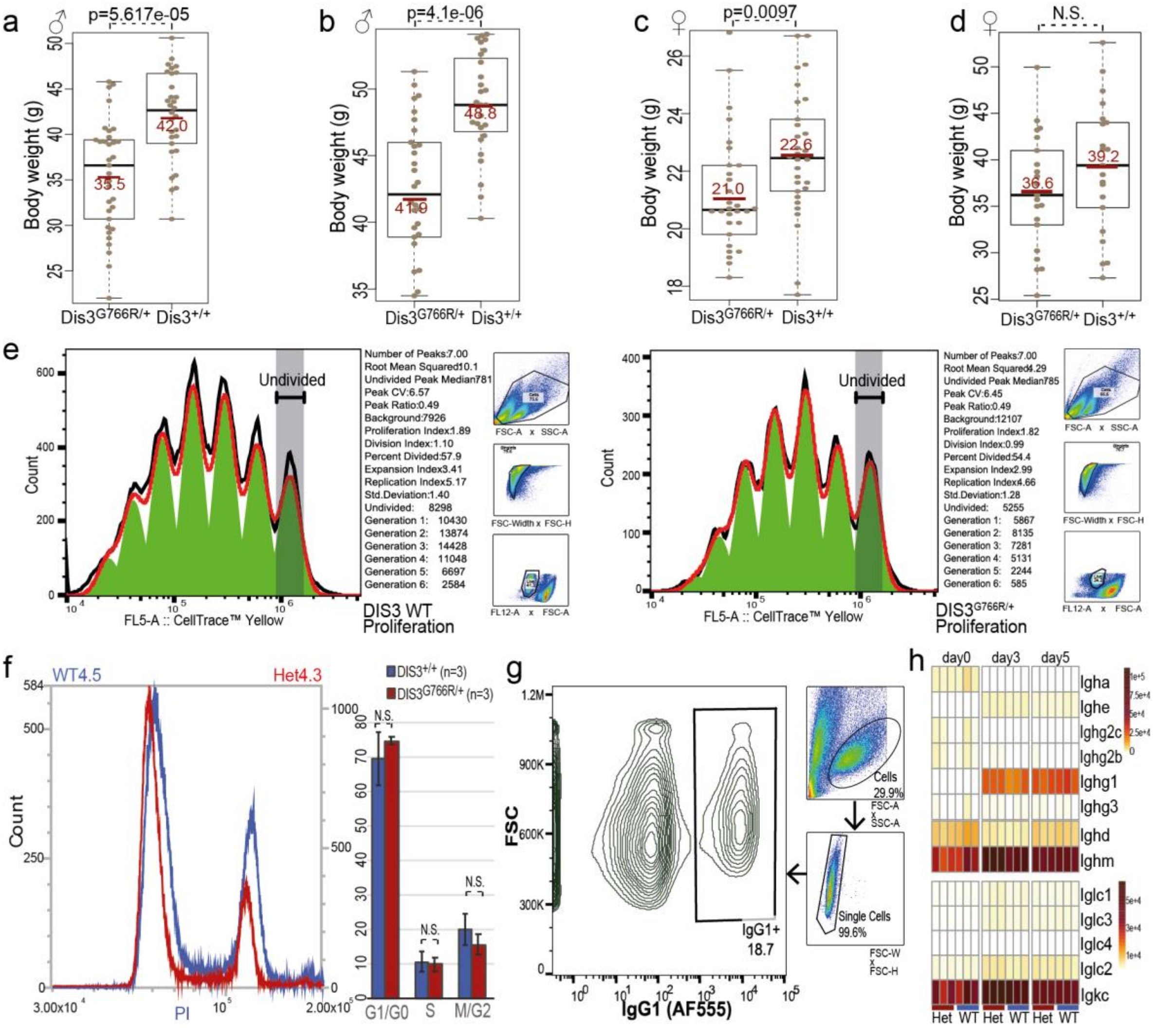

Supplementary Figure S3. Generalized growth phenotype of Dis3$^{G766R/+}$ knock-in mice. (A-D) Weight of 25-45 week male (A), 60-80 week male (B), 16-25 week female (C), and 25-40 week female **d** Dis3$^{G766R/+}$ mice compared with their Dis3$^{+/+}$ littermates. (Statistic: two-sided Wilcoxon rank-sum test.) **e** FlowJo proliferation tools analysis of representative Dis3$^{+/+}$ and DIS3$^{G766R/+}$ day 3 in vitro activated B-cell samples stained with CellTrace™ Yellow Cell proliferation assay. **f** Flow cytometric analysis of the cell cycle phase distribution estimating DNA content of MEF cells isolated from Dis3G766R/+ mice compared with their Dis3$^{+/+}$ littermates after 5 passages in cell culture, using propidium iodide (PI) staining. Quantification of mitotic phases in cells isolated from 3 litters of mice. (Statistic: two-sided t-test.)**g** Representative flow cytometry analysis of primary activated B-cells that exemplify the gating strategy for the CSR assay **h** No significant changes in frequencies of class switching in Dis3$^{+/+}$ and DIS3$^{G766R/+}$ day 0, day 3 and day 5 in vitro activated B-cells, as determined by the analysis of immunoglobulin heavy and light chain classes expression in RNA-seq experiments.

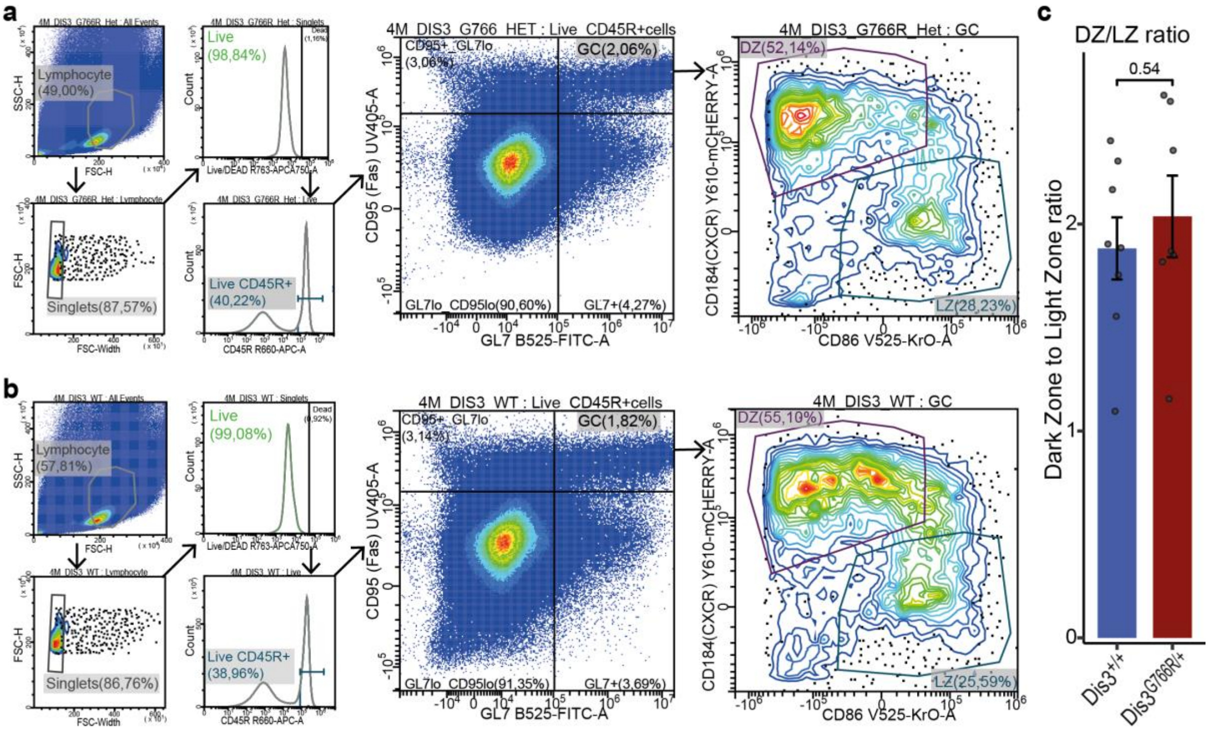

Supplementary Figure S4. Cytometric phenotype of splenic B-cells in Dis3$^{+/+}$ and Dis3$^{G766R/+}$ knock-in mice. (**a-b**) Representative flow cytometry analysis of primary speenocytes that exemplify the gating strategy. Splenic germinal center (GC) B-cells are defined as CD95 and GL7 positive, with dark zone (DZ) cells identified as CXCR4^{high} and CD86^{low} and light zone (LZ) as CXCR4^{low} CD86^{high}. **c** B-cells in Dis3$^{+/+}$ and Dis3$^{G766R/+}$ knock-in mice display comparable DZ:LZ ratios (~2) (Statistic: two-sided Wilcoxon rank-sum test.), error bars represent SEM points show results of tested individuals.

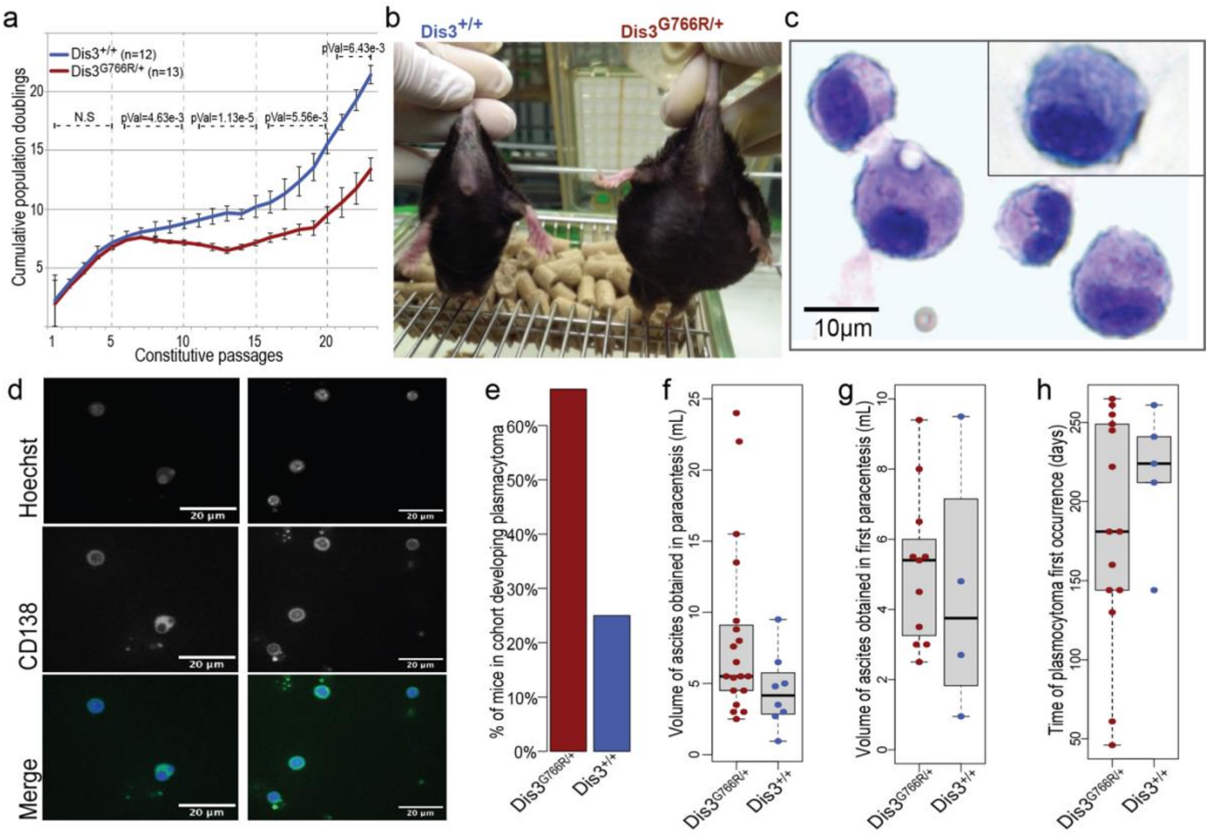

Supplementary Figure S5. Characterization of plasmacytomas that develop in Dis3$^{G766R/+}$ and WT mice. a Cumulative population doubling of WT and Dis3G766R/+ MEFs cultured according to the 3T3 protocol (Statistic: two-sided t-test.). **b** Example of Dis3$^{G766R/+}$ mouse that developed plasmacytoma compared with its Dis3$^{+/+}$ littermate. **c** Representative smear of ascites fluid, showing atypical plasma cells. **d** Representative immunofluorescent staining with α-CD138 antibody of atypical plasma cells smear in the ascites fluid. **e** Frequencies of plasmacytoma development in Dis3$^{G766R/+}$ mice and WT mice. **f** The volume (ml) of ascites that were collected during all paracentesis in Dis3$^{G766R/+}$ and Dis3$^{+/+}$ mice. **g** Volume (ml) of ascites that were collected during the first paracentesis in Dis3$^{G766R/+}$ and Dis3$^{+/+}$ mice. **h** Time of first plasmacytoma occurrence in mice that were treated with pristane. (Box plots show the median - center line, 25th–75th percentiles - box, whiskers to 1.5×IQR, and individuals as points)

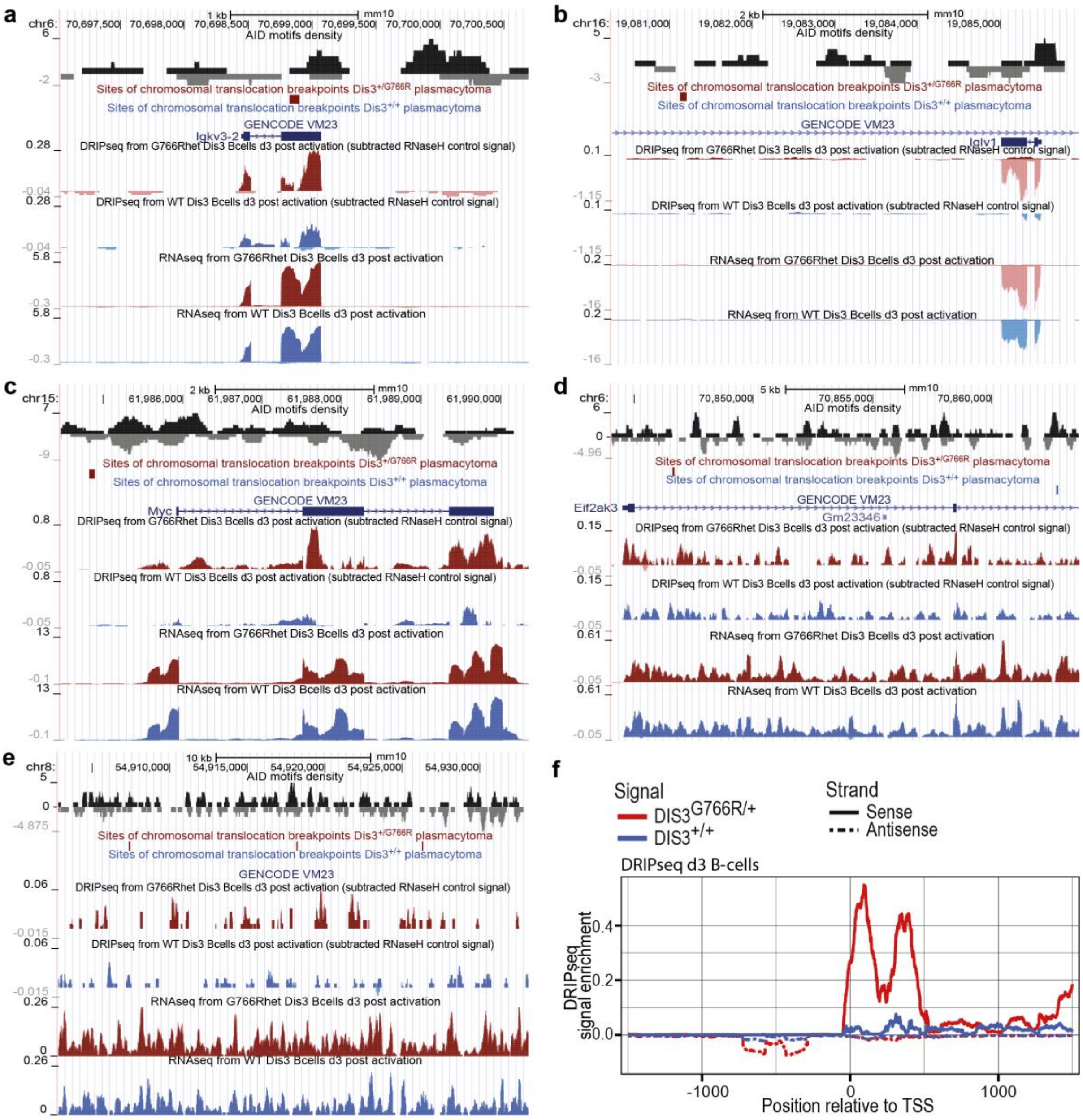

Supplementary Figure S6. Representative translocating genomic regions in murine plasmacytoma: **a** locus of immunoglobulin light chain kappa variable region; **b** locus of immunoglobulin light chain lambda variable region; **c** *Myc* locus; **d** *Ei2fak3* locus with high level intronic transcription; **e** a locus of unannotated intergenic transcription on chromosome 8 a with 3 inter-chromosomal breakpoints. DRIPseq signal is calculated by subtracting the corresponding RNaseH background control. **f** Meta-analyses of DRIP-seq signal over TSS of MM driver genes, in day 3 activated B-cells.

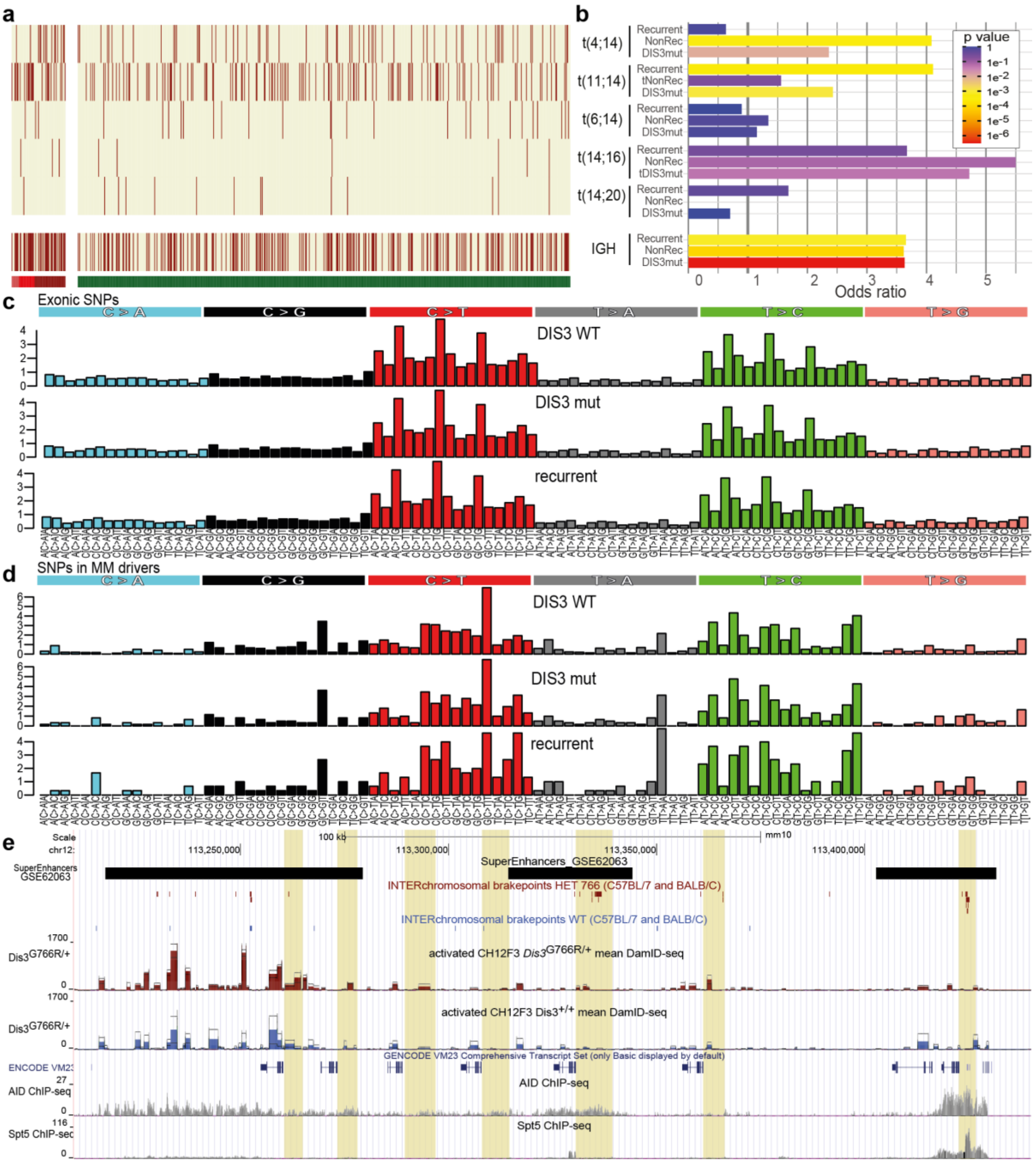

Supplementary Figure S7. Mutational footprint of DIS3 variants in MM patients. **a** Heatmap of MM driver translocations of the IGH locus in WT DIS3 CoMMpass patients *vs*. patients with a DIS3 mutation and their subset with recurrent DIS3 MM variant. **b** Barplot of the enrichment of the IGH MM-specific translocations in the groups' odds ratios of translocation occurrence in DIS3 mutant and WT patients. (Statistical significance was assessed using a two-sided Fisher's exact test.) **c** Mutational profiles of SNPs that were identified in whole exome sequencing (WES). **d** Mutational profiles of SNPs that were identified in WES in regions of known MM driver genes and their corresponding PROMPTs. **e** UCSC genome browser snapshot of the mouse IGH locus that shows $DIS3^{G766R}$ and $DIS3^{WT}$ occupancy over and in close vicinity of the switch regions, coinciding with AID and Spt5 occupancy and translocations that occur in plasmacytomas.

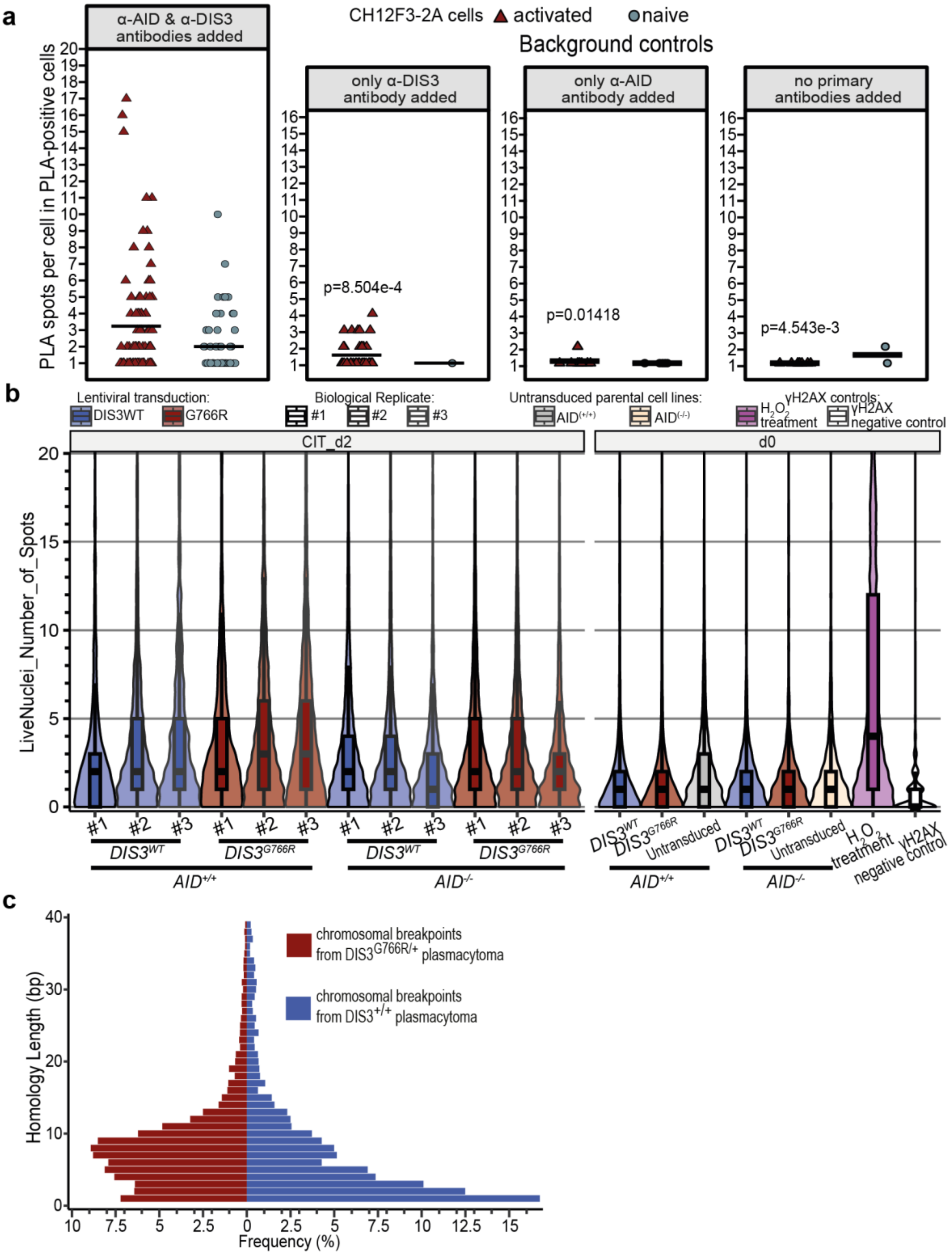

Supplementary Figure S8. DIS3–AID interaction and Dis3^{G766R}-dependent DNA damage in B cell. **a** Quantification of *in situ* PLA detection of DIS3 and AID interaction in CH12F3-2A as well as the signal in the 2 separate background controls performed. The number of interaction sites (PLA spots) in a cell was quantified and demonstrated to be significantly lower in background control which confirms the specificity of interaction. The background control giving the highest signal was chosen for further analysis for a most conservative approach. Analysis was restricted to cells with at least one PLA spot

identified (PLA-positive cells). Statistics were performed with the Wilcoxon test comparing given background control with the PLA signal for activated cells. The black horizontal lines represent means. **b** Quantification of high-throughput confocal imaging of DNA double-strand breaks, defined as the number of γ-H2AX foci per nucleus, in naïve and activated CH12F3-2A cells showed that DIS3^{G766R} expression led to elevated γ-H2AX levels in an AID-dependent manner. (Statistic: two-sided Wilcoxon rank-sum test.) **c** The distribution of microhomology lengths at chromosome breakpoints, showing slightly longer stretches in DIS3$^{G766R/+}$ cells (median 7 bp vs. 5 bp in WT).

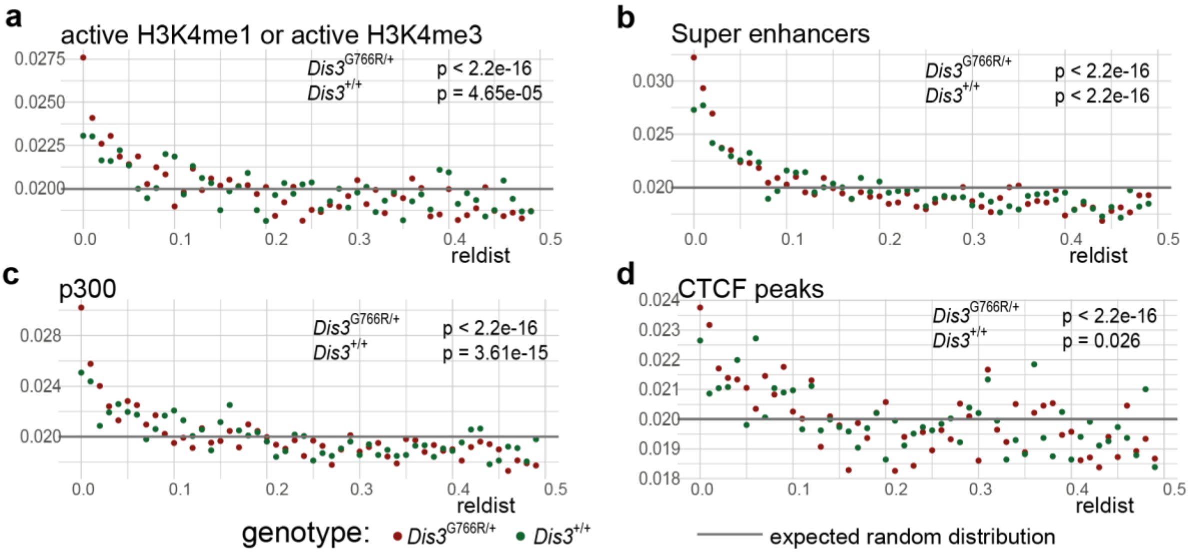

Supplementary Figure S9. Distribution analysis of translocation sites relative to active chromatin features. (a-d) Analysis of the distribution of relative distance of regions translocating in murine plasmacytomas to the ChIP-seq peaks active H3K4me1 or H3K4me3 (**a**) Super enhancers (**b**) p300 (**c**) and CTCF (**d**). Statistics were performed with the Pearson's χ^2 test comparing to observed relative distance frequencies to the expected random distribution.

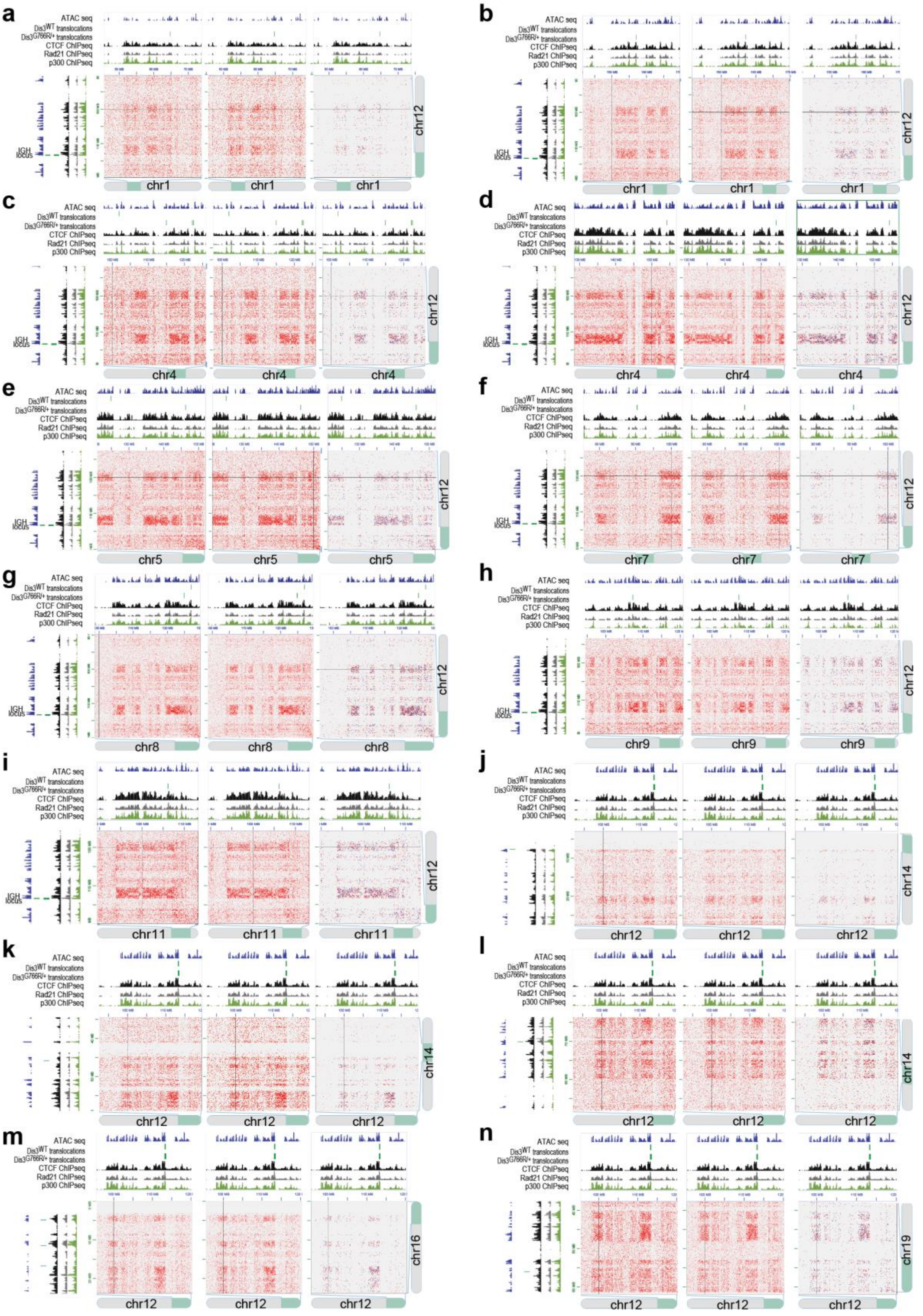

Supplementary Figure S10. The chromatin state of all regions translocating to the IGH locus in murine plasmacytoma of both genotypes. ATAC seq signal and CTCF, Rad21 and p300 occupancy

by ChIP seq as well as interchromosomal interactions detected using MicroC of regions translocating from the IGH locus to: chromosome 1(a,b), chromosome 4 (c,d), chromosome 5 (e), chromosome 7 (f), chromosome 8 (g), chromosome 9 (h), chromosome 11 (i), chromosome 14 (j,k,l), chromosome 16 (m) , chromosome 19 (n).

SUPPLEMENTARY TABLES

Supplementary Table 1

Primers (CH12F3.2A cell line generation):	
WT DIS3 cDNA F	aagtatatatttttaataagggtgggcgcgatgctcaggtccaagacgttc
WT DIS3 cDNA R	cactttgtacaagaaagctgggtcggcgcgcttctcaagcttcctcttc
site-directed mutagenesis F	catcactatCgcttagcctccccc
site-directed mutagenesis R	gggggaggctaagcGatagtgatg
mEGFP F	tccgaaaacctgtacttccaaggaaccggtatggtgagcaagggcgagg
mEGFP R	atcaccctgaaaatacaaattctcgctagccttgtacagctcgtccatgc
Primers (DamID-seq protocol):	
DpnI PCR	Nnnngtggtcgcggccgaggatc
Adaptor_top	ctaatacgactcactatagggcagcgtggtcgcggccgagga
DpnI adaptor bottom	Tcctcggccgcg
Y-adaptor top	Acactctttccctacacgacgctcttccgatct
Y-adaptor bottom	P-gatcggaagagcacacgtct (5′- phosphorylated)
P5- Illumina-2 PCR	aatgatacggcgaccaccgagatctacactctttccctacacgacgctcttccgatct
Primers (Dis3G766R/+ mice genotyping):	
766seq_F	gtctcaaaaacacacggcagagactc
766seq_FR	gaggatgtgtgagataagatacaccgg

DESCRIPTION OF SUPPLEMENTARY DATA

Supplementary Data S1. A bedpe format file with significant chromatin interactions from a MicroC experiment form DIS3$^{G766R/+}$ day3 activated B-cells.

Supplementary Data S2. A bedpe format file with significant chromatin interactions from a MicroC experiment form DIS3 WT day3 activated B-cells**.**

Supplementary Data S3–S6. The next four tables are provided in BEDPE format and contain structural variant (SV) translocations identified in plasmacytomas. All follow the same BEDPE structure:

1. chrom1 – chromosome of first breakpoint;
2. start1 – 0-based start of first region;
3. end1 – end of first region (cluster span);
4. chrom2 – chromosome of second breakpoint;
5. start2 – 0-based start of second region;
6. end2 – end of second region;
7. SVname – unique SV identifier (sequential ID + sample + SV type + additional annotation);
8. score – SVDetect weighted confidence after filtering (0–1, higher = stronger evidence);
9. strand1 – breakpoint 1 strand (".", undetermined);
10. strand2 – breakpoint 2 strand (".", undetermined);
11. NumberOfPairsAfterFiltering/previousNumberOfPairs – ratio of supporting discordant read pairs after vs. before filtering (e.g., 25/30).

Supplementary Data S3. A bedpe file format with DIS3$^{G766R/+}$ plasmacytoma translocations.

Supplementary Data S4. A bedpe file format with DIS3 WT plasmacytoma translocations.

Supplementary Data S5. A bedpe file format with DIS3$^{G766R/+}$ plasmacytoma translocations originating in the IGH locus.

Supplementary Data S6. A bedpe file format with DIS3 WT plasmacytoma translocations originating in the IGH locus.

Supplementary Data S7. A bed file with somatic single-nucleotide variants (SNVs) identified in DIS3$^{G766R/+}$ plasmacytomas, likely resulting from AID activity (C→N substitutions).

Supplementary Data S8. A bed file with somatic single-nucleotide variants (SNVs) identified in DIS3 WT plasmacytomas, likely resulting from AID activity (C→N substitutions).
