# Supplementary material for: DIS3 mutations enhance AID-driven translocations during B-cell activation, promoting transformation to multiple myeloma
Source: Nat Commun. 2026 Mar 14;17:3976. doi: 10.1038/s41467-026-70386-3 (PMC13133341; doi:10.1038/s41467-026-70386-3)
Supplement: Supplementary file 2 — Description of Additional Supplementary Files [file 41467_2026_70386_MOESM2_ESM.pdf]

Description of Additional Supplementary Files

Supplementary Data S1. A bedpe format file with significant chromatin interactions from a MicroC experiment form DIS3$^{G766R/+}$ day3 activated B-cells.

Supplementary Data S2. A bedpe format file with significant chromatin interactions from a MicroC experiment form DIS3 WT day3 activated B-cells.

Supplementary Data S3–S6. The next four tables are provided in BEDPE format and contain structural variant (SV) translocations identified in plasmacytomas. All follow the same BEDPE structure:

1. chrom1 – chromosome of first breakpoint;
2. start1 – 0-based start of first region;
3. end1 – end of first region (cluster span);
4. chrom2 – chromosome of second breakpoint;
5. start2 – 0-based start of second region;
6. end2 – end of second region;
7. SVname – unique SV identifier (sequential ID + sample + SV type + additional annotation);
8. score – SVDetect weighted confidence after filtering (0–1, higher = stronger evidence);
9. strand1 – breakpoint 1 strand (“.”, undetermined);
10. strand2 – breakpoint 2 strand (“.”, undetermined);
11. NumberOfPairsAfterFiltering/previousNumberOfPairs – ratio of supporting discordant read pairs after vs. before filtering (e.g., 25/30).

Supplementary Data S3. A bedpe file format with DIS3$^{G766R/+}$ plasmacytoma translocations.

Supplementary Data S4. A bedpe file format with DIS3 WT plasmacytoma translocations.

Supplementary Data S5. A bedpe file format with DIS3$^{G766R/+}$ plasmacytoma translocations originating in the IGH locus.

Supplementary Data S6. A bedpe file format with DIS3 WT plasmacytoma translocations originating in the IGH locus.

Supplementary Data S7. A bed file with somatic single-nucleotide variants (SNVs) identified in DIS3$^{G766R/+}$ plasmacytomas, likely resulting from AID activity (C→N substitutions).

Supplementary Data S8. A bed file with somatic single-nucleotide variants (SNVs) identified in DIS3 WT plasmacytomas, likely resulting from AID activity (C→N substitutions).
