# Supplementary material for: DIS3 mutations enhance AID-driven translocations during B-cell activation, promoting transformation to multiple myeloma
Source: Nat Commun. 2026 Mar 14;17:3976. doi: 10.1038/s41467-026-70386-3 (PMC13133341; doi:10.1038/s41467-026-70386-3)
Supplement: Supplementary file 11 — Reporting Summary [file 41467_2026_70386_MOESM11_ESM.pdf]

nature portfolio

Corresponding author(s): Andrzej Dziembowski, Tomasz Kuliński

Last updated by author(s): 10.10.2025

Reporting Summary

Nature Portfolio wishes to improve the reproducibility of the work that we publish. This form provides structure for consistency and transparency in reporting. For further information on Nature Portfolio policies, see our Editorial Policies and the Editorial Policy Checklist.

Statistics

For all statistical analyses, confirm that the following items are present in the figure legend, table legend, main text, or Methods section.

n/a	Confirmed	
☐	☒	The exact sample size (n) for each experimental group/condition, given as a discrete number and unit of measurement
☒	☐	A statement on whether measurements were taken from distinct samples or whether the same sample was measured repeatedly
☐	☒	The statistical test(s) used AND whether they are one- or two-sided<br>*Only common tests should be described solely by name; describe more complex techniques in the Methods section.*
☐	☒	A description of all covariates tested
☐	☒	A description of any assumptions or corrections, such as tests of normality and adjustment for multiple comparisons
☐	☒	A full description of the statistical parameters including central tendency (e.g. means) or other basic estimates (e.g. regression coefficient) AND variation (e.g. standard deviation) or associated estimates of uncertainty (e.g. confidence intervals)
☐	☒	For null hypothesis testing, the test statistic (e.g. F, t, r) with confidence intervals, effect sizes, degrees of freedom and P value noted<br>*Give P values as exact values whenever suitable.*
☒	☐	For Bayesian analysis, information on the choice of priors and Markov chain Monte Carlo settings
☒	☐	For hierarchical and complex designs, identification of the appropriate level for tests and full reporting of outcomes
☐	☒	Estimates of effect sizes (e.g. Cohen's d, Pearson's r), indicating how they were calculated

Our web collection on statistics for biologists contains articles on many of the points above.

Software and code

Policy information about availability of computer code

Data collection	*Provide a description of all commercial, open source and custom code used to collect the data in this study, specifying the version used OR state that no software was used.*
Data analysis	Bioinformatic analysis was done applying: R Project for Statistical Computing (v. 3.6.0); STAR split read aligner (v2.7.4a); samtools (v1.10); bedtools (v2.29.2); Cutadapt (v. 1.18); NxTrim (v0.4.3.0); Subread (v2.0); BWtool (v1.0 gamma); DESeq2 (v. 1.22).<br>FlowJo v10.8.1; ImageJ.

For manuscripts utilizing custom algorithms or software that are central to the research but not yet described in published literature, software must be made available to editors and reviewers. We strongly encourage code deposition in a community repository (e.g. GitHub). See the Nature Portfolio guidelines for submitting code & software for further information.

Data

Policy information about availability of data

All manuscripts must include a data availability statement. This statement should provide the following information, where applicable:

- Accession codes, unique identifiers, or web links for publicly available datasets
- A description of any restrictions on data availability
- For clinical datasets or third party data, please ensure that the statement adheres to our policy

The sequencing data discussed in this publication have been deposited in NCBI's Gene Expression Omnibus and are accessible through GEO Series accession

number: GSE155631.
The mass spectrometry proteomics data have been deposited to the ProteomeXchange Consortium via the PRIDE repository with the dataset identifier PXD050438.

Research involving human participants, their data, or biological material

Policy information about studies with human participants or human data. See also policy information about sex, gender (identity/presentation), and sexual orientation and race, ethnicity and racism.

Reporting on sex and gender	*Use the terms sex (biological attribute) and gender (shaped by social and cultural circumstances) carefully in order to avoid confusing both terms. Indicate if findings apply to only one sex or gender; describe whether sex and gender were considered in study design; whether sex and/or gender was determined based on self-reporting or assigned and methods used. Provide in the source data disaggregated sex and gender data, where this information has been collected, and if consent has been obtained for sharing of individual-level data; provide overall numbers in this Reporting Summary. Please state if this information has not been collected. Report sex- and gender-based analyses where performed, justify reasons for lack of sex- and gender-based analysis.*
Reporting on race, ethnicity, or other socially relevant groupings	*Please specify the socially constructed or socially relevant categorization variable(s) used in your manuscript and explain why they were used. Please note that such variables should not be used as proxies for other socially constructed/relevant variables (for example, race or ethnicity should not be used as a proxy for socioeconomic status). Provide clear definitions of the relevant terms used, how they were provided (by the participants/respondents, the researchers, or third parties), and the method(s) used to classify people into the different categories (e.g. self-report, census or administrative data, social media data, etc.) Please provide details about how you controlled for confounding variables in your analyses.*
Population characteristics	*Describe the covariate-relevant population characteristics of the human research participants (e.g. age, genotypic information, past and current diagnosis and treatment categories). If you filled out the behavioural & social sciences study design questions and have nothing to add here, write "See above."*
Recruitment	*Describe how participants were recruited. Outline any potential self-selection bias or other biases that may be present and how these are likely to impact results.*
Ethics oversight	*Identify the organization(s) that approved the study protocol.*

Note that full information on the approval of the study protocol must also be provided in the manuscript.

Field-specific reporting

Please select the one below that is the best fit for your research. If you are not sure, read the appropriate sections before making your selection.

☒ Life sciences ☐ Behavioural & social sciences ☐ Ecological, evolutionary & environmental sciences

For a reference copy of the document with all sections, see nature.com/documents/nr-reporting-summary-flat.pdf

Life sciences study design

All studies must disclose on these points even when the disclosure is negative.

Sample size	The sample size is provided on figures and figures descriptions.
Data exclusions	Not applicable
Replication	All replicates of experiments yielded comparable results.
Randomization	Not applicable
Blinding	Not applicable

Reporting for specific materials, systems and methods

We require information from authors about some types of materials, experimental systems and methods used in many studies. Here, indicate whether each material, system or method listed is relevant to your study. If you are not sure if a list item applies to your research, read the appropriate section before selecting a response.

Materials & experimental systems

n/a	Involved in the study	
☐	☒	Antibodies
☐	☒	Eukaryotic cell lines
☒	☐	Palaeontology and archaeology
☐	☒	Animals and other organisms
☒	☐	Clinical data
☒	☐	Dual use research of concern
☒	☐	Plants

Methods

n/a	Involved in the study	
☐	☒	ChIP-seq
☐	☒	Flow cytometry
☒	☐	MRI-based neuroimaging

Antibodies

Antibodies used

anti-Mouse IgG1 Invitrogen Cat. No. A-21127, RRID:AB_2535769
anti-AID Invitrogen Cat. No. PA5-18913, RRID:AB_10978944
anti-DIS3 Proteintech Cat. No. 14689-1-AP RRID:AB_2091025
anti-γH2A.X Abcam Cat. No. ab2893, RRID:AB_303388
anti-CD45R/B220–APC clone RA3-6B2, BD Biosciences, Cat. #553092; RRID:AB_398531
anti-CD95–BUV395 clone Jo2, BD Biosciences, Cat. #740254; RRID:AB_2739999
anti-GL7– CoraLite® Plus 488 clone GL-7, Proteintech Cat No. CL488-65261; RRID:AB_3084186
anti-CD184 (CXCR4) Monoclonal Antibody (clone 2B11), Invitrogen eBioscience™, Cat. #14-9991-82; RRID:AB_842770
anti-CD86– BD Horizon™ BV510 clone GL1, BD Biosciences Cat. #563077; RRID:AB_2737991
anti-γH2AX (phospho S139)-PerCP-eFluor 710; Invitrogen, 46-9865-42; RRID:AB_2573918

Validation

anti-Mouse IgG1 Invitrogen https://www.thermofisher.com/antibody/product/Goat-anti-Mouse-IgG1-Cross-Adsorbed-Secondary-Antibody-Polyclonal/A-21127
anti-AID Invitrogen https://www.thermofisher.com/antibody/product/AID-Antibody-Polyclonal/PA5-18913
anti-DIS3 Proteintech https://www.ptglab.com/products/DIS3-Antibody-14689-1-AP.htm?srsltid=AfmBOoqoFohVGGtYym7JmhJ2AuSEHYnWC01cPAG8CM1TDY3Kc0fcqzrx
anti-γH2A.X https://www.abcam.com/en-us/products/primary-antibodies/gamma-h2ax-phospho-s139-antibody-ab2893?srsltid=AfmBOorG07QfHEZXLjn8bmoVsVKIntuyTLbDSScQnHMaSMl9mE517mo_
anti-CD45R/B220 https://www.bdbiosciences.com/en-pl/products/reagents/flow-cytometry-reagents/research-reagents/single-color-antibodies-ruo/apc-rat-anti-mouse-cd45r-b220.553092?tab=product_details
anti-CD95 https://www.bdbiosciences.com/en-pl/products/reagents/flow-cytometry-reagents/research-reagents/single-color-antibodies-ruo/buv395-hamster-anti-mouse-cd95.740254?tab=antibody_details
anti-GL7 https://www.ptglab.com/products/GL7-Antibody-CL488-65261.htm?srsltid=AfmBOophM_KE_ZFzmpluLv6dmc10m-OYeMUKYkN7JZyX-HPpFAgNw15g
anti-CD184 (CXCR4) https://www.thermofisher.com/antibody/product/CD184-CXCR4-Antibody-clone-2B11-Monoclonal/14-9991-82
anti-CD86 https://www.bdbiosciences.com/en-pl/products/reagents/flow-cytometry-reagents/research-reagents/single-color-antibodies-ruo/bv510-rat-anti-mouse-cd86.563077?tab=product_details
anti-γH2AX (phospho S139)-PerCP-eFluor 710; https://www.thermofisher.com/antibody/product/Phospho-Histone-H2A-X-Ser139-Antibody-clone-CR55T33-Monoclonal/46-9865-42

Eukaryotic cell lines

Policy information about cell lines and Sex and Gender in Research

Cell line source(s)

CH12F3 murine B-cell lymphoma cell line Ricken BRC cell bank
CH12F3-2A AID$^{-/-}$ B-cell lymphoma cell line (Kerafast, Cat# ESP013, RRID:CVCL_GZ21) was obtained from Kerafast

Authentication

The cell line was authenticated based on its characteristic morphology and its ability to be activated by supplementation with 1 µg/ml anti-CD40 antibody, 5 ng/ml IL-4, and 1 ng/ml TGF-β1.

Mycoplasma contamination

All cells were regulary tested for mycoplasma contamination

Commonly misidentified lines
(See ICLAC register)

not applicable

Animals and other research organisms

Policy information about studies involving animals; ARRIVE guidelines recommended for reporting animal research, and Sex and Gender in Research

Laboratory animals

C57BL/6J Institut clinique de la souris (ICS) Illkirch, France;
BALB/CanNCrlCmd Mossakowski Medical Research Institute, Warsaw, Poland;
C57BL/6J-BS-K706-tm1c Institut clinique de la souris (ICS) Illkirch, France IR00003831;
C57BL/6J-Dis3em1Iimcb/Tar This paper Dis3em1Iimcb/Tar

Wild animals	not applicable
Reporting on sex	Sex-based analyses of the mice used in this study were not performed. Based on existing knowledge and the nature of the experiments, no sex-based differences are expected, and there is no current evidence to suggest that sex would influence the outcomes observed.
Field-collected samples	not applicable
Ethics oversight	All procedures were approved by the First Local Ethical Committee in Warsaw affiliated at the University of Warsaw, Faculty of Biology (approval numbers WAW/092/2016, WAW/177/2016, WAW/642/2018). Housing in animal facilities was performed in conformity with local and European Commission regulations under the control of veterinarians and with the assistance of trained technical personnel.

Note that full information on the approval of the study protocol must also be provided in the manuscript.

Plants

Seed stocks	*Report on the source of all seed stocks or other plant material used. If applicable, state the seed stock centre and catalogue number. If plant specimens were collected from the field, describe the collection location, date and sampling procedures.*
Novel plant genotypes	*Describe the methods by which all novel plant genotypes were produced. This includes those generated by transgenic approaches, gene editing, chemical/radiation-based mutagenesis and hybridization. For transgenic lines, describe the transformation method, the number of independent lines analyzed and the generation upon which experiments were performed. For gene-edited lines, describe the editor used, the endogenous sequence targeted for editing, the targeting guide RNA sequence (if applicable) and how the editor was applied.*
Authentication	*Describe any authentication procedures for each seed stock used or novel genotype generated. Describe any experiments used to assess the effect of a mutation and, where applicable, how potential secondary effects (e.g. second site T-DNA insertions, mosiacism, off-target gene editing) were examined.*

ChIP-seq

Data deposition

☒ Confirm that both raw and final processed data have been deposited in a public database such as GEO.

☒ Confirm that you have deposited or provided access to graph files (e.g. BED files) for the called peaks.

Data access links<br>*May remain private before publication.*	Publicly available data from the following publications were used:<br>ChIP-seq AID SRA: SRP003605;<br>ChIP-seq H3K4me3, H3K4me1, H3K27ac, p300, CTCF and Rad21 SRA: SRP075985
Files in database submission	*Provide a list of all files available in the database submission.*
Genome browser session<br>(e.g. UCSC)	http://genome.ucsc.edu/cgi-bin/hgTracks?db=hg19&hubUrl=http://212.87.21.131/users/tkulinski/Dis766_Bcells/Dis3_766_Bcell_Avr.txt

Methodology

Replicates	AID, CTCF and Rad21 were prepared in triplicates whereasthe rest were prepared in a single replicate
Sequencing depth	*Describe the sequencing depth for each experiment, providing the total number of reads, uniquely mapped reads, length of reads and whether they were paired- or single-end.*
Antibodies	anti-AID - Rabbit polyclonal described in Chaudhuri et al., Nature (2003)
Peak calling parameters	*Specify the command line program and parameters used for read mapping and peak calling, including the ChIP, control and index files used.*
Data quality	*Describe the methods used to ensure data quality in full detail, including how many peaks are at FDR 5% and above 5-fold enrichment.*
Software	ChIP-seq reads were aligned to the mouse reference genome (GRCm38.p6) using bowtie2. All replicates were pooled and used to call peaks with MACS2 (v2.2.7.1) using IGG control as the background.

Flow Cytometry

Plots

Confirm that:

☒ The axis labels state the marker and fluorochrome used (e.g. CD4-FITC).

☒ The axis scales are clearly visible. Include numbers along axes only for bottom left plot of group (a 'group' is an analysis of identical markers).

☒ All plots are contour plots with outliers or pseudocolor plots.

☒ A numerical value for number of cells or percentage (with statistics) is provided.

Methodology

Sample preparation	1–2 × 10^6 splenocytes were incubated with Live/Dead Fixable Near-IR dye (Invitrogen, L34975; 1:1000 dilution) for 30 min at room temperature, protected from light. Cells were then blocked with anti-mouse CD16/CD32 (Fc block, 1 µg per 10^6 cells; BD Biosciences) or purified mouse IgG (1–2 µg per 10^6 cells) in PBS containing 1% BSA for 15 min on ice. Without washing out the blocking reagent, fluorochrome-conjugated antibodies were added at the manufacturer's recommended concentrations in PBS + 1% BSA and incubated for 30 min at 4 °C, protected from light.
Instrument	CytoFLEX LX Flow Cytometer (Beckman Coulter)
Software	CytExpert (v2.23, Beckman Coulter)
Cell population abundance	*Describe the abundance of the relevant cell populations within post-sort fractions, providing details on the purity of the samples and how it was determined.*
Gating strategy	Dead cells and doublets were excluded by standard gating. GC B cells were identified as $B220^+$ $GL7^+$ $CD95^+$. Within this population, dark zone (DZ) and light zone (LZ) subsets were defined as follows:<br>• DZ B cells: CXCR4high CD86low<br>• LZ B cells: CXCR4low CD86high

☒ Tick this box to confirm that a figure exemplifying the gating strategy is provided in the Supplementary Information.
